# Glycosphingolipid expression in squamous cell carcinoma of the upper aerodigestive tract

**DOI:** 10.1016/S1808-8694(15)30030-6

**Published:** 2015-10-19

**Authors:** Marcilio Ferreira Marques Filho, Fernando Walder, Helio K. Takahashi, Luciana L. Guimarães, Ameria K. Tanaka, Onivaldo Cervantes, Anita H. Straus

Correction of image subtitles

**Table:** Relation between the means of total GSL quantities compared to the normal mucosa of the upper aerodigestive tract.

Statistics	SCC *µ*g GSL/mg Tissue	Mucosa *µ*g GSL/mg Tissue	t test (p)
Mean	3,57	1,92	
Standard deviation	2,35	1,54	0,001 *
n	33	33	

This article has received corrections in agreement with the ERRATUM published in Volume 72 Number 5.

**Figure 1:**
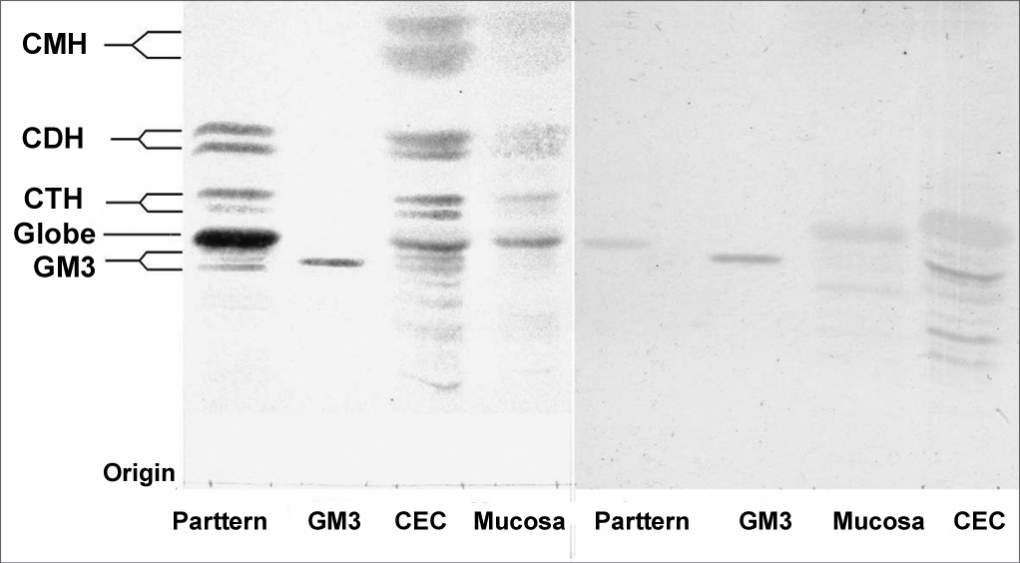
HPTLC slide dyed by orcinol/H2SO4. (left) and (right) HPTLC dyed by resorcinol. Pattern – Erythrocyte GLS pattern. GM3 pattern (Sigma®). SCC - GSLs of SCC. Mucosa - GSLs of normal mucosa.

**Chart 1:**
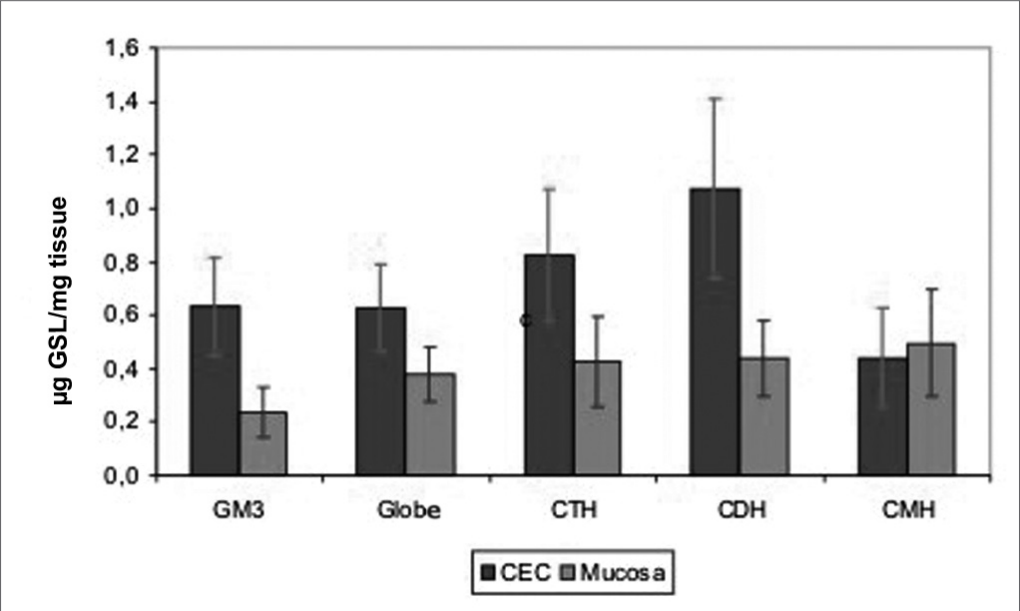
GSL quantity average in SCC and in the mucosa of the upper aerodigestive tract

**Figure 2:**
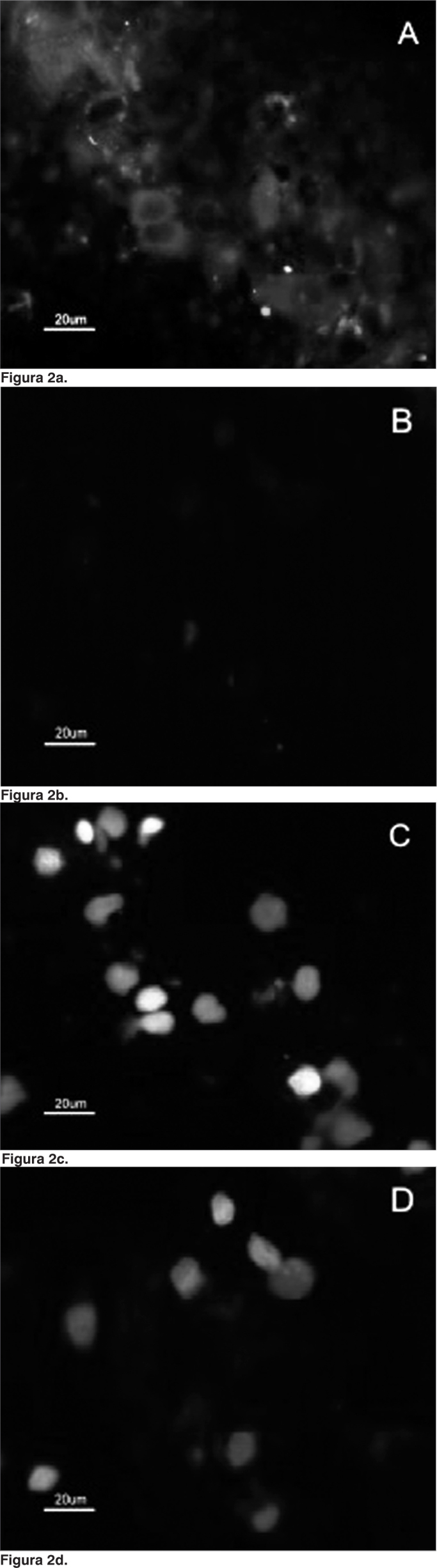
Indirect immunefluorescence microphotography where we can see the reactivity difference of SCC cells when compared to that of the normal mucosa cells at MoAb DH2 (AntiGM3). A. SCC cells dyed with DH2 (green). B. Normal mucosa cells marked with DH2 (green). C. SCC cell nuclei dyed with DAPI (blue). D. Nuclei of the normal mucosa cells dyed with DAPI (blue)

**Figure 3:**
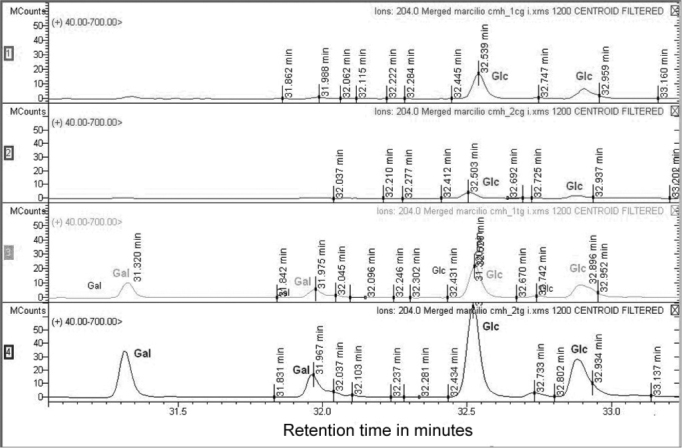
CMH mass spectrometry. The upper lines (A and B) show the identification of sugar residue in the CMH fraction from the upper aerodigestive tract mucosa. Lines C and D correspond to the identification of sugar residues in the SCC CMH fraction. Gal – galactose, Glc – glucose

**Figure 4:**
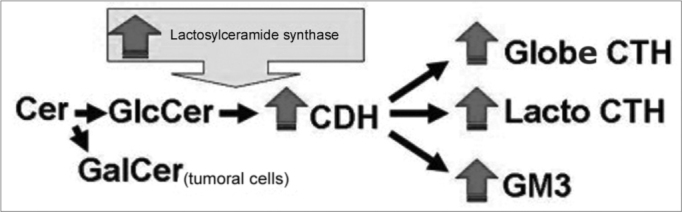
Proposed scheme for the synthesis of gangliosides in order to explain the increase in GSL expression in SCC of the upper aerodigestive tract. The greater activity of lactosilceramide synthase would increase GM3, CTH and CDH expression and, consequently, reduce Glc supply for the synthesis of GlcCer; thus the SCC cells would need to use Gal in order to produce GalCer and maintain the cell membrane structure.

